# Evaluation of a Multisite Safe Infant Sleep Education and Crib Distribution Program

**DOI:** 10.3390/ijerph18136956

**Published:** 2021-06-29

**Authors:** Trina C. Salm Ward, Terri J. Miller, Iman Naim

**Affiliations:** 1 Helen Bader School of Social Welfare, University of Wisconsin-Milwaukee, Milwaukee, WI 53211, USA; 2 Health Protection Division, Georgia Department of Public Health, Atlanta, GA 30303, USA; terri.miller@dph.ga.gov (T.J.M.); imannaim7@gmail.com (I.N.)

**Keywords:** health promotion, infant mortality prevention, sudden unexpected death in infancy, sudden infant death syndrome, social and cultural determinants, priority populations

## Abstract

Rates of sleep-related infant deaths have plateaued in the past few decades despite ongoing infant sleep practice recommendations to reduce risk of sleep-related infant deaths by the American Academy of Pediatrics. The state department of public health trained facilitators at 28 sites across the state to facilitate a group safe sleep educational program. A prospective, matched pre- and post-test cohort design with follow-up was used to evaluate changes in self-reported knowledge, intentions, and practices. The final sample included 615 matched pre- and post-test surveys, and 66 matched follow-up surveys. The proportion of correct responses on all knowledge and intended practice items increased significantly from pre- to post-test. When asked where their babies would have slept if they had not received the portable crib, 66.1% of participants planned to use a recommended sleep location (e.g., crib or bassinet). At post-test, 62.3% planned to change something about their infant’s sleep based on what they learned. At follow-up, knowledge was maintained for all but two items and practices and for half of practice items. The results suggest that participating in the education program was associated with increased knowledge and intended adherence, but that these changes were not maintained at follow-up. These results are in line with the research literature that finds a difference in intentions and actual practices after the baby is born.

## 1. Introduction

In the United States, 3600 infants die annually of sleep-related causes such as sudden infant death syndrome (SIDS), unknown causes, and accidental suffocation and strangulation [1]. While sleep-related infant death rates decreased in the 1990s, they have plateaued more recently [2]. The American Academy of Pediatrics (AAP) Taskforce on SIDS recommends the following practices to reduce the risk of sleep-related infant deaths: back sleep position; firm sleep surface; room-sharing without bed-sharing; avoiding soft bedding and overheating; avoiding smoke, alcohol, or illicit drug exposure; breastfeeding; routine immunizations; and offering a pacifier [2].

In the state of Georgia, USA, the leading causes of death in the post-neonatal period are SIDS and suffocation [3]. The rates of SIDS for non-Hispanic black infants are disproportionately higher than for non-Hispanic white infants, with rates of 3.6 deaths per 1000 live births and 2.4, respectively [3]. Recent child fatality review data indicate that among 152 sleep-related infant deaths, 55.9% occurred in an adult bed, 54.6% occurred while sharing a sleep surface, and 45.4% were found on their stomachs [4]. A survey of 10,231 mothers in Georgia also found that risky sleep practices were common: 40% of mothers reported their infant slept in a non-supine position and 67% shared a sleep surface with their infant [5].

The literature on safe sleep interventions has demonstrated some success in increasing knowledge and changing practices to reduce risk of sleep-related infant deaths, but has also called for more rigorous evaluation designs [6,7]. The Georgia Department of Public Health (DPH) developed and pilot-tested a group safe sleep education program in Fulton County. This pilot-test occurred in a controlled setting—at one county health department and delivered by three master’s-prepared state health department health educators [8]. The evaluation results suggested that participating in the program was associated with significant improvements in participant knowledge of safe sleep recommendations and intentions to adhere to recommendations [8]. Follow-up surveys indicated retention of knowledge and reported adherence to recommended safe sleep practices [8].

Indig and colleagues [9] suggest that “to achieve a population-wide health improvement, public health interventions found effective in selected samples need to be ‘scaled up’ and implemented more widely.” In this context, “scaling up” can refer to implementing a program into a broader community or less-controlled settings [9]. In line with this definition, DPH aimed to increase safe sleep knowledge, intentions, and practices across the state by “scaling up” the program. To achieve this goal, DPH trained staff at social service and health agencies across the state to facilitate the safe sleep education program at their local agencies. 

The purpose of this study was to assess program participant self-reported knowledge, intentions, and practices before and after participating in the program.

## 2. Materials and Methods

### 2.1. Design

A prospective, matched pre- and post-test cohort design with a follow-up survey was used to evaluate changes in self-reported knowledge, intentions, and practices among program participants [10]. 

### 2.2. Program Description

DPH created a group safe infant sleep education and crib distribution program which was based on the National Institute of Child Health and Human Development’s Safe to Sleep^®^ public education campaign materials [11], which are based on the AAP recommendations for a safe infant sleeping environment [2]. Program development was guided by the Health Belief Model [12]. The Health Belief Model posits that an individual’s behavior is reliant upon their perceptions about the health behavior, specifically, perceived susceptibility to harm, perceived severity of the harm, perceived benefit of practicing the behavior, perceived barriers to practicing the behavior, cues to action, and self-efficacy [12]. These concepts were incorporated into the program, for example, by describing susceptibility and severity (e.g., “three infants a week die of sleep-related causes in our state”), benefit of practices (e.g., “reduced risk of death”), and addressing potential barriers to adherence (e.g., concerns about choking or infant comfort). Sessions were structured to include “ask the parent” questions to help parents demonstrate self-efficacy (i.e., understanding of the concepts). Detailed information on program development has been published elsewhere [8]. The program consisted of a one-time educational session (approximately 30-45 minutes) using a PowerPoint presentation that included talking points for the session facilitator. At the end of the education session, participants received a portable crib—which also served as a cue to action—and instructions for and demonstration of crib set-up and take-down.

The program was initially piloted by three trained health educators in one county; pilot results demonstrated increased safe sleep knowledge, intentions, and practices [8]. Using these findings, DPH refined the program to reinforce and add components, for example, based on recent literature on accidental bed-sharing [13,14], a slide was added addressing feeding at night [15]. 

To “scale up” the intervention, DPH invited local agencies across the state to be trained to provide the safe sleep education program at their agency. Prospective facilitators at participating agencies completed a DPH-provided training webinar. The webinar lasted approximately 60 minutes and included: a description of the impetus for the education program (including data on sleep-related infant death rates in the state); current AAP recommendations to reduce the risk of sleep-related infant deaths [2]; results of the pilot project; evaluation design requirements (pre- and post-test surveys); and instructions for facilitating the program and distributing the portable cribs. After agency representatives completed the webinar, DPH shipped surveys and cribs to sites, and provided ongoing phone and in person consultation on program facilitation.

### 2.3. Participants

DPH invited the following types of agencies to participate: local social service agencies, health departments, clinics, and other organizations that served expectant and new parents; sites were recruited via an email invitation from the DPH safe infant sleep program manager. Interested sites completed an application form that outlined program expectations, including participating in the webinar, hosting education sessions, administering and maintaining confidentiality of surveys, securely storing portable cribs, and returning completed surveys to DPH. No specific credentials or educational background were required of session facilitators—the only requirement was that they completed the training webinar prior to facilitating sessions, and that they agreed to promote the AAP recommendations for safe sleep [2]. Thirty-eight sites in counties with the highest infant mortality rates were selected to participate and completed the mandatory webinar.

Sites invited program participants who met the following eligibility criteria: (1) women close to, or in, their third trimester of pregnancy or a close family member of the expectant woman, and (2) demonstrated financial need (based on receipt of services through one of the following: Women, Infants, and Children Nutritional Program; Temporary Assistance for Needy Families; Medicaid; or Supplemental Nutrition Assistance Program), or had otherwise been identified as in need of assistance with obtaining a crib. 

### 2.4. Procedures

At the education sessions, facilitators gave participants a survey packet that included a consent cover letter that outlined participants’ rights regarding participation, and the pre-test and post-test surveys. Participants kept the cover letter for their records; if they chose not to participate, they discarded the survey. Participants completed the pre-test survey before the education session was delivered and the post-test survey after completion of the education session. Follow-up survey procedures included a question in the pre-test survey that asked if participants were willing to be contacted for a follow-up survey. If they agreed, they entered their contact information and estimated due date. For participants who agreed to be contacted, the DPH team waited until several weeks after the due date had passed, then sent an electronic survey link and also attempted to complete follow-up surveys via telephone. No monetary incentives were offered for completing surveys. The study was conducted in accordance with the Declaration of Helsinki, and the protocol was reviewed and determined exempt by the DPH Institutional Review Board (protocol #180902) on 9 September 2018.

### 2.5. Measures

Survey questions were developed by the research team and drawn from previous studies [8,16,17,18]. Pre-test surveys included an informed consent cover letter and 19 questions about demographic data, intended infant care practices, and knowledge of safe sleep recommendations. Post-test surveys included 14 questions on intended practices, knowledge of recommendations, and open-ended questions about impressions of the education program. The follow-up survey also included questions about nighttime feeding locations and frequency of unintentionally falling asleep while feeding [13]. Each survey took approximately 10–15 min to complete.

### 2.6. Data Analysis

Knowledge, intention, and practice items were coded as dichotomous variables (correct versus incorrect) based on AAP recommendations [2]. Univariate comparisons between pre- and post-test items and between post-test and follow-up items were made using McNemar’s Chi square tests [19]. The Statistical Package for the Social Sciences (IBM SPSS, Version 25.0, Armonk, NY, USA) was used for all data analysis and a *p* value of <0.05 was considered statistically significant. Potential differences in sample demographics across sites were assessed via Pearson’s Chi square tests.

## 3. Results

Of the 38 sites that were initially invited to participate, 28 participated during the project period, facilitating a total of 192 educational sessions across the state (sessions ranged from 1 to 19 participants). All 28 sites designated one primary educator who facilitated the education sessions as well as support staff who assisted with session logistics, such as scheduling, check-in, and distributing surveys and cribs. Surveys were collected from 621 participants, and 66 follow-up surveys were completed. Six participant surveys were excluded from analysis because they were missing a paired post-test. The resulting sample consisted of 615 matched pre- and post-test surveys, and 66 matched follow-up surveys. The majority of participants (94.5%) identified as expectant mothers (see Table 1 for demographics). Participants primarily identified as non-Hispanic (89.5%), and African American (48.1%), or white (46.4%), with an average age of 26.9 years (*Standard Deviation [SD]* = 6.9). Almost half of participants (45.8%) had a high school diploma or less. Over three-quarters (84.7%) reported using Medicaid for insurance. Slightly over half (59.9%) identified as single; 40.1% were married or in a relationship. At follow-up, average infant age was 13.1 weeks (*SD* = 6.5). When examining demographic characteristics by site, significant differences were found across all demographic variables (race, ethnicity, education level, health insurance used, relationship status, and age).

### 3.1. Knowledge of Safe Sleep Recommendations

At post-test, a significantly larger proportion of respondents correctly answered all eleven knowledge items than at pre-test (see Table 2), suggesting an increased knowledge of safe sleep recommendations at post-test. The largest changes in the proportion of correct responses from pre- to post-test occurred for the following items: “pacifier decreases risk” (increased by 39.4%), and “recommended back position” (increased by 24.1%).

At follow-up, no significant differences were found between respondents correctly answering all but two knowledge items. Significant decreases in the proportion of correct responses were found for “pacifier decreases risk” and “back sleeping doesn’t increase choking risk” suggesting that fewer participants retained knowledge about these two items over time.

### 3.2. Practices

At post-test, a larger proportion of respondents correctly answered all items (see Table 3). The largest changes in the proportion of correct responses from pre- to post-test occurred for the following items: “sleep environment (no soft items)” (increased by 32.5%), “sleep location (only recommended surfaces)” (increased by 27.3%). 

At follow-up, no significant differences were found for the following items: “own crib,” “room-sharing,” and “smoking.” A significantly smaller proportion of respondents correctly answered the following practice items at follow-up: “back only position,” “recommended sleep location” and “breastfeeding,” suggesting that adherence to these recommended practices declined. 

### 3.3. Additional Findings

Post-test surveys included a question about where participants’ babies would have slept if they had not received the portable crib. Of 551 respondents who answered the question, 66.1% planned to use a recommended sleep location (such as a crib, bassinet, or portable crib). The remainder of participants planned to purchase or borrow an item later (15.8%), to use an adult bed (12.9%), or other location (sitting device, floor, 1.5%), or did not know where baby would have slept (3.8%).

The post-test survey also asked, “Based on what you learned, will you change anything with your baby’s sleep?” (yes/no response choices). Almost two-thirds of respondents (62.3%) reported that they planned to change something based on what they learned. The respondents were also asked to specify what they would change. Of the 356 written responses, the three main themes were: place baby on back to sleep (30.3%), no soft items in the sleep environment (23.9%), and no bed-sharing/baby will sleep alone (19.4%).

The post-test also assessed program helpfulness based on a 5-point Likert-type scale; the majority of respondents rated the program as helpful (*Mean [M]* = 4.93, *SD* = 0.45). At post-test, 188 participants responded to the open-ended question, “Is there anything else you would like us to know?” All responses were highly positive, noting the program was helpful and informative, and expressing gratitude for receipt of the portable crib. Among the written comments, several respondents noted that the educational session corrected misinformation about infant sleep recommendations; for example, “I thought babies could not sleep on their back” (see Table 4 for example comments). 

At follow-up, respondents were asked about usual nighttime feeding location. Among 66 respondents, 50.8% reported “on an adult bed,” and 49.2% reported “on a chair, recliner, or sofa.” Most participants (71.2%) reported never falling asleep while feeding at night, while 18.2% reported rarely falling sleep, 7.6% sometimes, 1.5% often, and 1.5% always. Among the 33 respondents who reported nighttime feeding in an adult bed, 60.6% reported never falling asleep in that location, and among the 32 respondents who reported nighttime feeding on a chair, recliner, or sofa, 81.3% reported never falling asleep in that location.

## 4. Discussion

The results demonstrated that at post-test, a larger proportion of program participants correctly responded regarding knowledge of infant safe sleep recommendations, and that their intended infant sleep practices better aligned with safe sleep recommendations. These results suggest the current mode of delivery (a group educational program using PowerPoint presentation materials) is appropriate for this target population of expectant parents experiencing financial need. The results also suggest that the facilitator training was effective in “scaling up” the program across multiple sites in the state. 

A common limitation of many safe sleep intervention studies is that they do not include a follow-up survey to determine longer-term retention of knowledge and practices [6,7]. Data from the limited number of follow-up surveys suggest only partial retention of knowledge and adherence to safe sleep recommendations. These results are in line with research literature that suggests that despite knowledge and intentions regarding recommended safe sleep practices, families experience difficulties in maintaining practices over time (e.g., once baby is home and the family experiences sleep challenges) [8,17,20,21,22,23]. Potential ways to address these difficulties in maintaining knowledge and practices over time may be to offer a “booster” session after the infant is born, with the aims of: (1) reinforcing knowledge of recommendations, and (2) helping address challenges to adherence to safe sleep recommendations. Potential delivery modes could be through a group format, one-on-one phone calls, or mailed written materials. Continued pilot-testing and evaluation of the booster sessions would ensure that the session addresses the most salient concerns for parents and to assess appropriateness of the mode of delivery.

Respondents’ written comments added clarity regarding potential program benefits. Specifically, a few respondents reported that prior to the education program, they had understood that supine sleeping was appropriate (even recommended) for young infants. Placing an infant supine to sleep on a separate sleep surface reduces infants’ risk of death by as much as 50% [2]. Moreover, at the time of attending the program, almost half of respondents demonstrated some need for a sleep surface, either because they did not currently have a recommended sleep surface, intended to use another location, or did not know where baby would have slept. After attending this education session, all participants had access to at least one recommended sleep surface (the portable crib offered at the education session). While it can be difficult to quantify the amount of reduction of risk or potential lives saved through such a program, these results suggest that for some families, risk of sleep-related infant deaths may have been reduced by participating in this program and correcting misinformation. 

Indig and colleagues note that promising public health interventions need to be “scaled up” to achieve population-wide health improvement, suggesting four primary stages of scaling up: (1) program development (including the theoretical basis); (2) pilot-testing to determine program efficacy; (3) field testing or implementation of a “real world” trial of the program across multiple settings and locations; and (4) dissemination at the population-level [9]. DPH’s efforts align well with these stages – DPH: (1) developed the program guided by the Health Belief Model [12]; (2) pilot-tested the program in a controlled setting (with three highly trained health educators in one county) [8]; and (3) field-tested the program through delivery by local facilitators across 28 sites. The results of this field-testing process demonstrated improvements in knowledge and intended practices at post-test in the larger sample; however, we did not assess fidelity of program implementation by site. For future programs looking to “scale up” a promising public health intervention, we recommend building in resources to assess program fidelity, for example, by randomly observing sessions across sites using a fidelity checklist to score adherence to key program components, and providing this feedback to session facilitators.

This evaluation has some limitations. Participants were included if they had a demonstrated financial need, thus, the results may not be generalizable to populations with varying income levels. Our evaluation design also cannot rule out alternative explanations for changes in knowledge and practice [10]. It is possible that participants responded to self-report questions in a socially desirable manner versus their true intentions, a common limitation in survey research [5,16,17], especially given the stigma associated with not adhering to safe sleep recommendations [24,25]. The very low response rate for the follow-up survey limits this study’s ability to draw strong conclusions regarding whether knowledge and practices were maintained over time; offering monetary incentives to complete the follow-up survey could increase willingness to participate. An additional limitation of this evaluation is that program fidelity was not assessed, which would have helped determine if the education program was being carried out as intended at all sites. Further, our evaluation did not collect cost data regarding delivering this intervention, thus, we are unable to determine if the effects and cost outweigh the potential benefit of this program. Future studies might also consider adding additional risk-related variables, such as antenatal care. Despite these limitations, this study contributes to the growing body of evaluation literature on infant safe sleep interventions, and adds to the scientific literature on implementing and “scaling up” promising public health interventions.

## 5. Conclusions

The results suggest that participating in the education program was associated with significant increases in knowledge and intentions regarding infant safe sleep recommendations at post-test. The follow-up survey results suggest adding a “booster” session to reinforce knowledge and help families address barriers to adhering to safe sleep recommendations. Continued rigorous evaluation and follow-up efforts are needed to ensure effectiveness of such a component. The recommended framework for “scaling up” a promising public health intervention was a useful guide for this public health effort [9]. Future studies should build in robust designs for conducting follow-up surveys and fidelity monitoring to further strengthen results and identify opportunities to improve program implementation.

## Figures and Tables

**Table 1:** Demographic characteristics.

Characteristic		Pre-Test ^1^ (*n* = 615)		Follow-Up (*n* = 66)	
		Number ^2^	%	Number	%
Relationship to baby	Mother	567	94.5	62	96.9
	Father	15	2.5	2	3.1
	Grandparent	10	1.7	0	0
	Other family	5	0.8	0	0
	Friend	3	0.5	0	0
Race	Black/African American	289	48.1	33	51.6
	White/Caucasian	279	46.4	27	42.2
	Multi-Racial	21	3.5	3	4.7
	Other	12	2.0	1	1.6
Ethnicity	Not Hispanic	531	89.5	55	85.9
	Hispanic	62	10.5	9	14.1
Age (years), mean (*SD*)	26.9 (6.9)			27.6 (5.8)	
Education level	Some high school	73	12.1	8	12.3
	High school graduate/GED	203	33.7	14	21.5
	Some college/technical school	180	29.9	24	36.9
	2-year college/tech graduate	59	9.8	8	12.3
	4-year college graduate	66	10.9	9	13.8
	Postgraduate/professional degree	22	3.8	2	31
Health insurance	Medicaid	508	84.7	54	83.1
	Private insurance	66	11.0	7	10.8
	Other	3	0.5	0	0
	No insurance	23	3.8	4	6.2
Relationship status	Single	359	59.9	40	60.6
	Married/in a relationship	240	40.1	26	39.4
Infant age (weeks), mean (*SD*)		Not applicable		13.1 (6.5)	

^1^ Demographic data, except for infant age, were collected at pre-test only. ^2^ Missing data: relation to baby (*n* = 15), race (*n* = 14), ethnicity (*n* = 22), age (*n* = 11), education (*n* = 12), insurance (*n* = 15), relationship status (*n* = 16).

**Table 2:** Knowledge—comparison of correct responses on matched surveys.

Knowledge Items (Correct Response)	Pre-Test ^1^ (*n* = 615) Number (%)	Post-Test ^2^ (*n* = 615) Number (%)	Pre-Test–Post-Test *p* Value ^3^	Post-Test (*n* = 66) Number (%)	Follow-Up ^4^ (*n* = 66) Number (%)	Post-Test—Follow-Up *p* Value ^3^
What is recommended sleep position? (back only)	438 (71.5)	586 (95.6)	<0.001	64 (97.0)	65 (98.5)	1.000
What is recommended about where baby should sleep? (room-share, separate surface)	456 (74.9)	585 (96.1)	<0.001	62 (93.9)	57 (86.4)	0.227
Soft items increase risk (true)	548 (90.4)	591 (97.5)	<0.001	64 (98.5)	63 (96.9)	1.000
Breastmilk decreases risk (true)	527 (87.7)	581 (96.7)	<0.001	63 (95.5)	63 (95.5)	1.000
Side or stomach sleep increases risk (true)	442 (72.8)	574 (94.6)	<0.001	65 (98.5)	65 (98.5)	1.000
Pacifier decreases risk (true)	255 (42.8)	490 (82.2)	<0.001	57 (86.4)	41 (62.1)	<0.001
Too much clothing/blankets increase risk (true)	534 (88.4)	584 (96.7)	<0.001	64 (97.0)	63 (95.5)	1.000
Flat, firm surface decreases risk (true)	502 (83.3)	570 (94.5)	<0.001	62 (95.4)	63 (96.9)	1.000
Couch sleeping increases risk (true)	507 (82.7)	594 (96.9)	<0.001	65 (98.5)	62 (93.9)	0.250
Back sleeping doesn’t increase choking risk (true)	398 (67.9)	524 (89.4)	<0.001	64 (97.0)	51 (77.3)	0.002
Smoke exposure increases risk (true)	544 (91.6)	579 (97.5)	<0.001	66 (100)	62 (93.9)	NS

^1^ Pre-test missing data: position (*n* = 1), location (*n* = 5), soft items (*n* = 5), breastmilk (*n* = 11), side/stomach (*n* = 6), pacifier (*n* = 12), clothing (*n* = 6), flat firm surface (*n* = 7), couch sleeping (*n* = 2), choking (*n* = 26), smoke (*n* = 19). ^2^ Post-test missing data: position (*n* = 1), location (*n* = 1), soft items (*n* = 4), breastmilk (*n* = 4), side/stomach (*n* = 4), pacifier (*n* = 8), clothing (*n* = 5), flat firm surface (*n* = 6), couch (*n* = 0), choking (*n* = 4), smoke (*n* = 2). ^3^ Results of McNemar’s chi-square tests. ^4^ No missing data for follow-up survey.

**Table 3:** Intended and actual practices—comparison of correct responses on matched surveys.

Practices	Correct Response	Pre-Test ^1^ (*n* = 615) Number (%)	Post-Test ^2^ (*n* = 615) Number (%)	Pre-Test–Post-Test *p* Value ^3^	Post-Test (*n* = 66) Number (%)	Follow-Up ^5^ (*n* = 66) Number (%)	Post-Test—Follow-Up *p* Value ^3^
Sleep position	Back only	488 (80.4)	587 (96.7)	<0.001	62 (93.9)	52 (78.8)	0.013
Sleep location	Recommended surfaces	366 (65.5)	519 (92.8)	<0.001	61 (93.8)	41 (63.1)	<0.001
Sleep environment	No soft items	337 (64.1)	508 (96.6)	<0.001	63 (100.0)	52 (82.5)	^6^
Own crib (always, no bed-sharing)		335 (54.9)	544 (89.2)	<0.001	57 (86.4)	51 (77.3)	0.238
Room-sharing		566 (92.8)	588 (96.4)	0.004	63 (95.5)	61 (92.4)	0.727
Breastfeeding		485 (79.8)	495 (81.4)	0.006	55 (88.7)	25 (40.3)	<0.001
Maternal smoking (not endorsed) ^4^					59 (89.4)	58 (87.9)	1.000
Others’ smoking (not endorsed) ^4^					55 (83.3)	56 (84.8)	1.000

^1^ Pre-test missing data: position (*n* = 5), location (*n* = 7), environment (*n* = 38), own crib (*n* = 4), room-share (*n* = 5), breastfeed (*n* = 6). ^2^ Post-test missing data: position (*n* = 3), location (*n* = 50), environment (*n* = 60), own crib (*n* = 1), room-share (*n* = 0), breastfeed (*n* = 2). ^3^ Results of McNemar’s chi-square tests. ^4^ Smoke exposure measured at pre-test but not post-test; results are reported for matched pre-test and follow-up surveys. ^5^ Follow-up missing data: position (*n* = 0), location (*n* = 0), environment (*n* = 0), own crib (*n* = 0), room-share (*n* = 0), breastfeed (*n* = 3), maternal smoke (*n* = 0), other smoke (*n* = 2). ^6^ Unable to determine significance due to less than required responses in one cell.

**Table 4:** Example written comments regarding corrected misinformation from participants.

Topic	Example Quotes
Back sleeping	“I thought babies couldn’t sleep on their back.”
	“I was taught an infant should sleep on the stomach or side before this class.”
	“I was always taught for a baby to lay on the stomach after feeding to prevent choking but today I have learned that being on the back is the safest position.”
No bed-sharing	“I thought the baby was supposed to sleep in the same bed until 3 months old.”
	“I will have my baby sleep alone. I often thought it was okay for babies to sleep in bed with you.”
	“I was planning on cosleeping but I would just like to have a separate sleep surface next to the bed.”
	“Baby was going to be sleeping in bed with me and a 2 year old but learning the potential dangers, he has his own sleep space.”
Pacifier use	“I didn’t know that sleeping with a pacifier was okay.”
	“Wasn’t sure about pacifiers but they are okay.”
No soft items	“Debating ordering crib bumpers—will not.”
No sitting device	“I thought the car seat was okay but now I know to immediately remove her and put her in the crib at home.”

## Data Availability

The datasets generated for this study are available on request to the corresponding author.
